# Supplementary material for: Analysis of nearly 3000 archaeal genomes from terrestrial geothermal springs sheds light on interconnected biogeochemical processes
Source: Nat Commun. 2024 May 14;15:4066. doi: 10.1038/s41467-024-48498-5 (PMC11094006; doi:10.1038/s41467-024-48498-5)
Supplement: Supplementary file 1 — Supplementary Information [file 41467_2024_48498_MOESM1_ESM.pdf]

Supplementary information

Analysis of nearly 3,000 archaeal genomes from terrestrial geothermal springs sheds light on interconnected biogeochemical processes

Qi *et al.*

The PDF file includes:

Supplementary Figs. S1 to S12

Supplementary Table 1 and 2

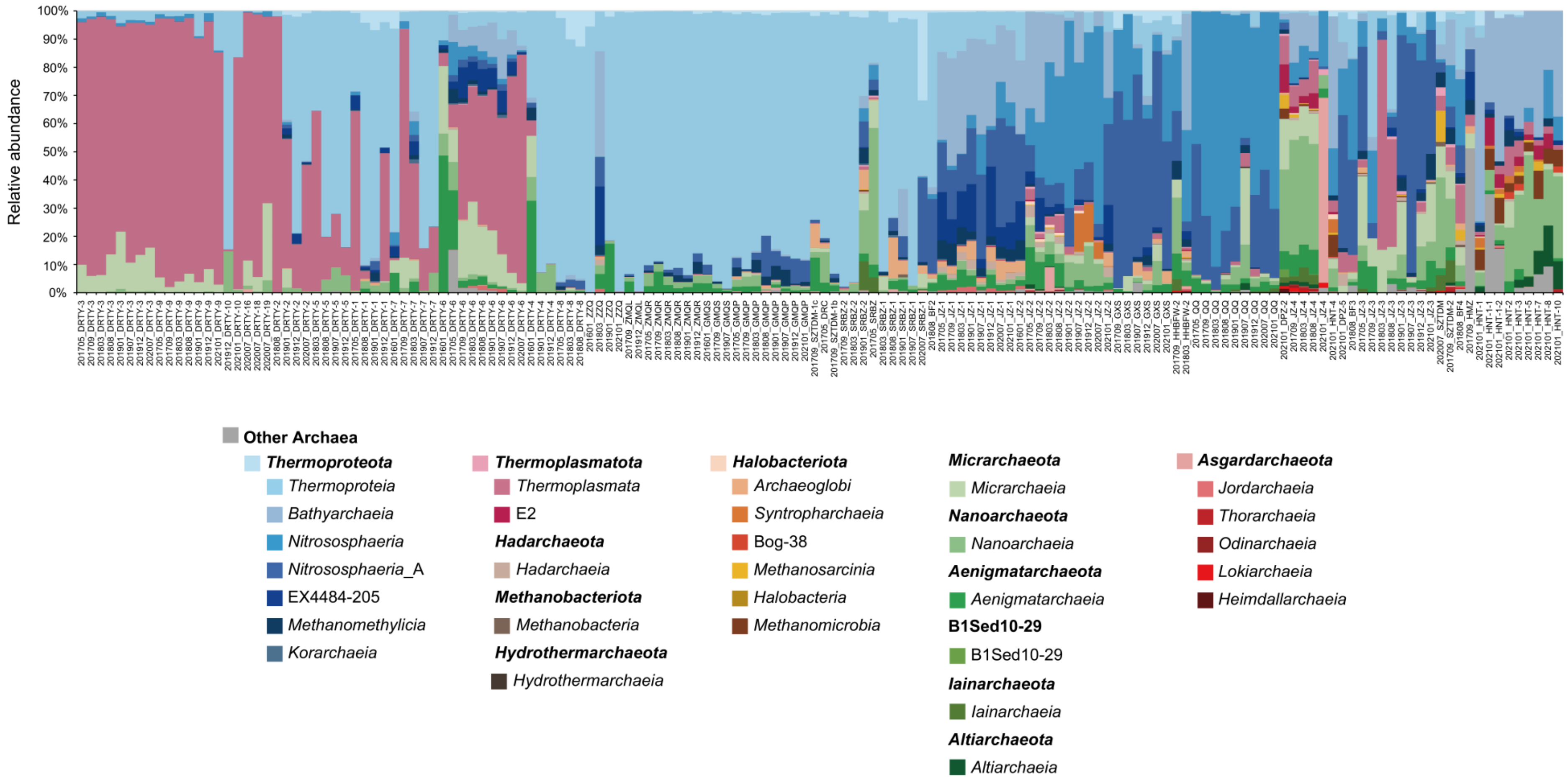

Supplementary Fig. 1 Diversity of archaeal classes across 152 geothermal springs and their relative abundance in archaeal communities based on *rpS3* genes.

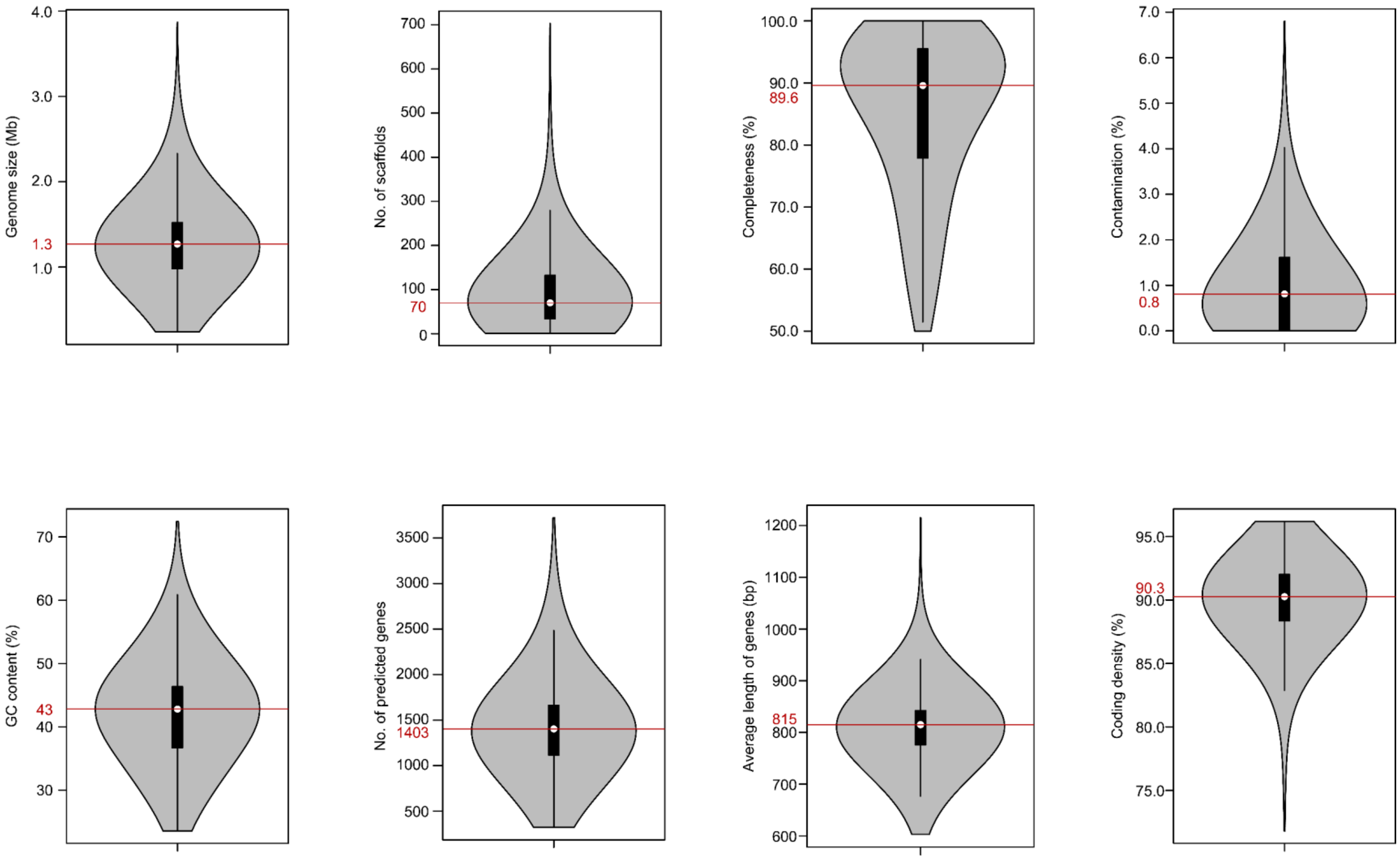

Supplementary Fig. 2 Basic genomic features of 2,949 archaeal MAGs reconstructed in this study.

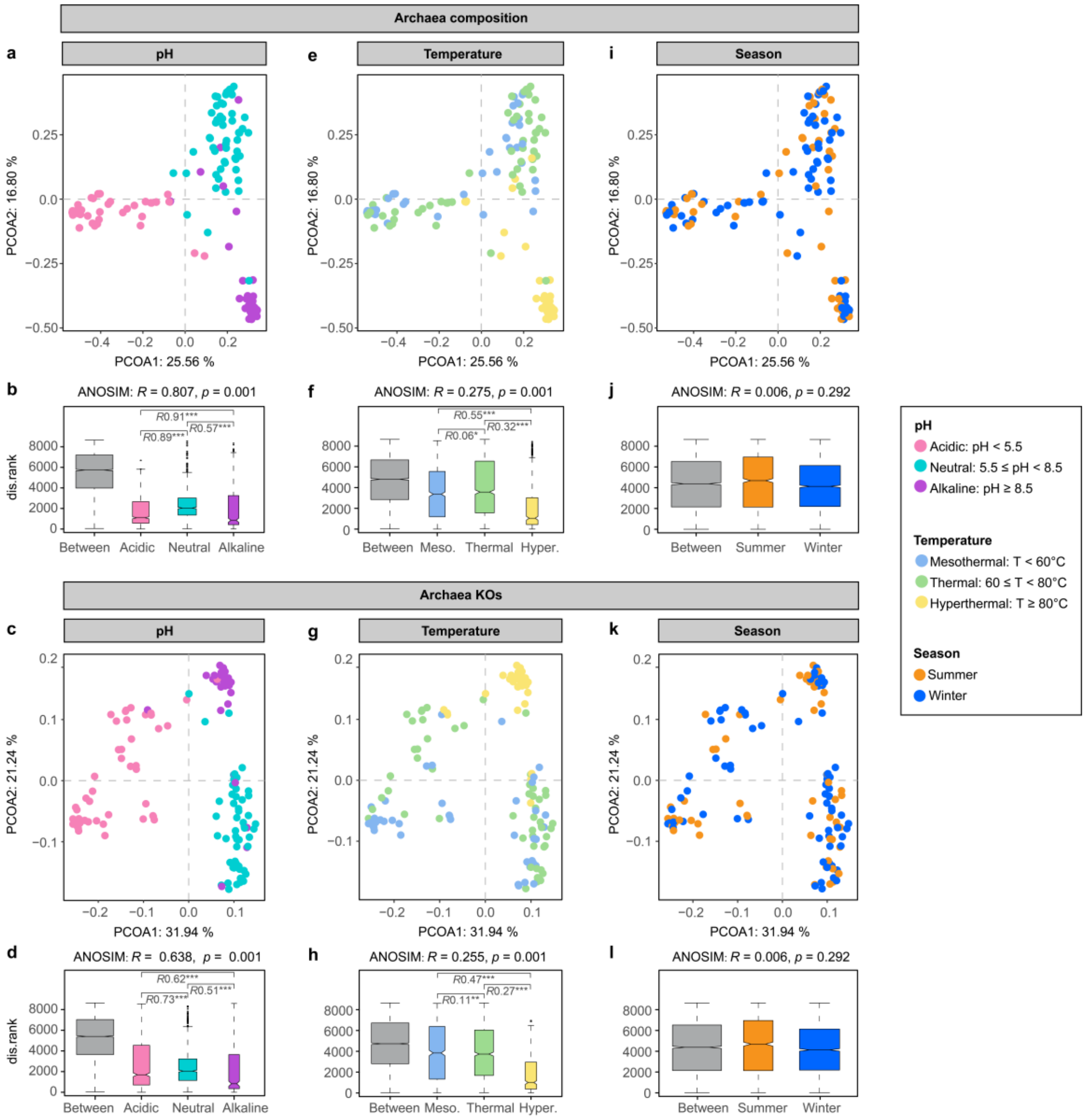

Supplementary Fig. 3 Ordination of archaeal communities in geothermal springs. PCoA clustering and ANOSIM tests of archaeal composition (a, b) and function (c, d) at different pH; PCoA clustering and ANOSIM tests of archaeal composition (e, f) and function (g, h) at different temperatures; PCoA clustering and ANOSIM tests of archaeal composition (i, j) and function (k, l) in different sampling seasons.

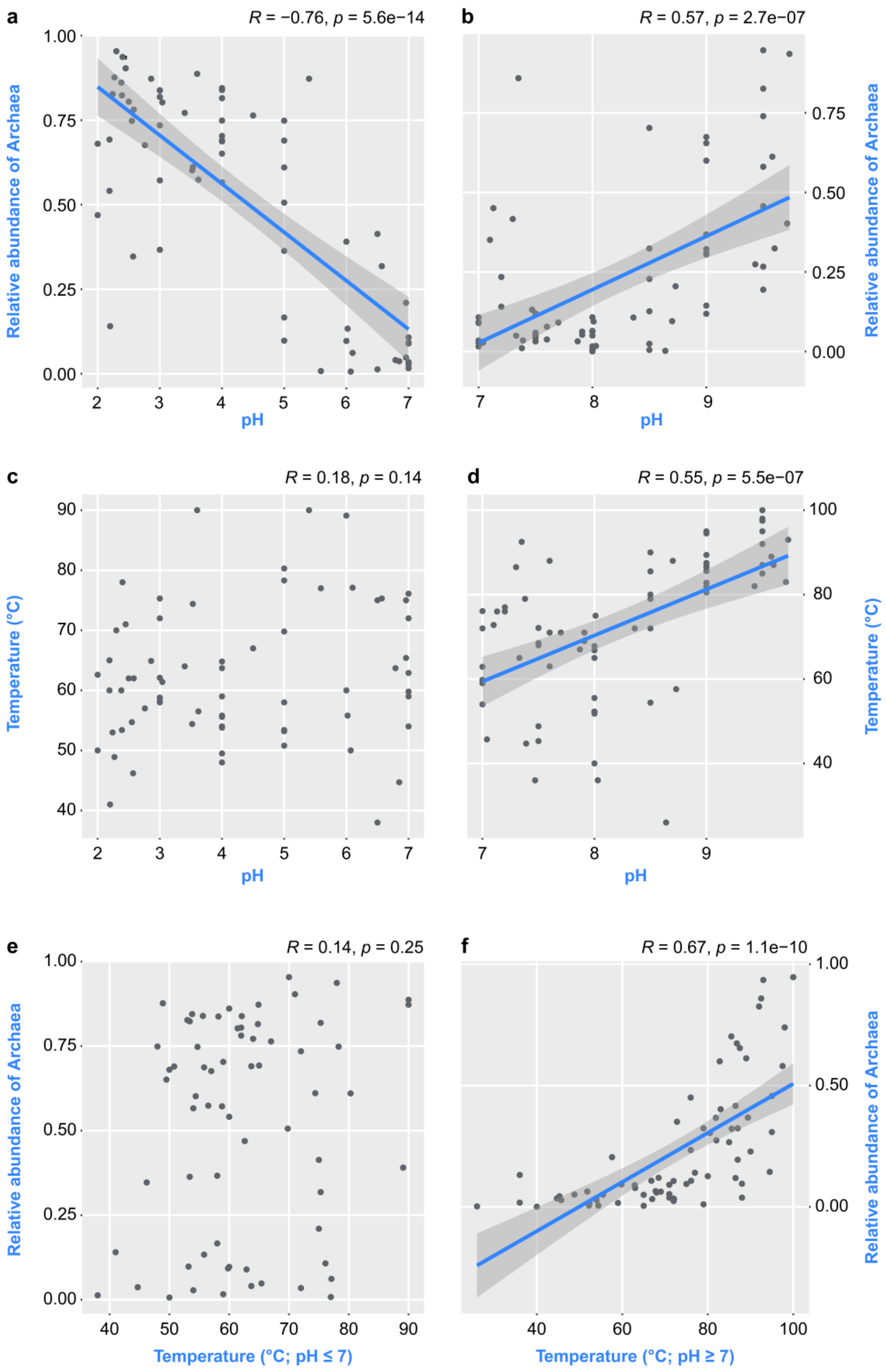

Supplementary Fig. 4 The correlation between relative abundance of Archaea and acidic pH (a) and alkaline pH (b); the correlation between temperature and acidic pH (c) and alkaline pH (d) in geothermal springs; the correlation between relative abundance of Archaea and temperature in acidic geothermal springs (e) and alkaline geothermal springs (f).

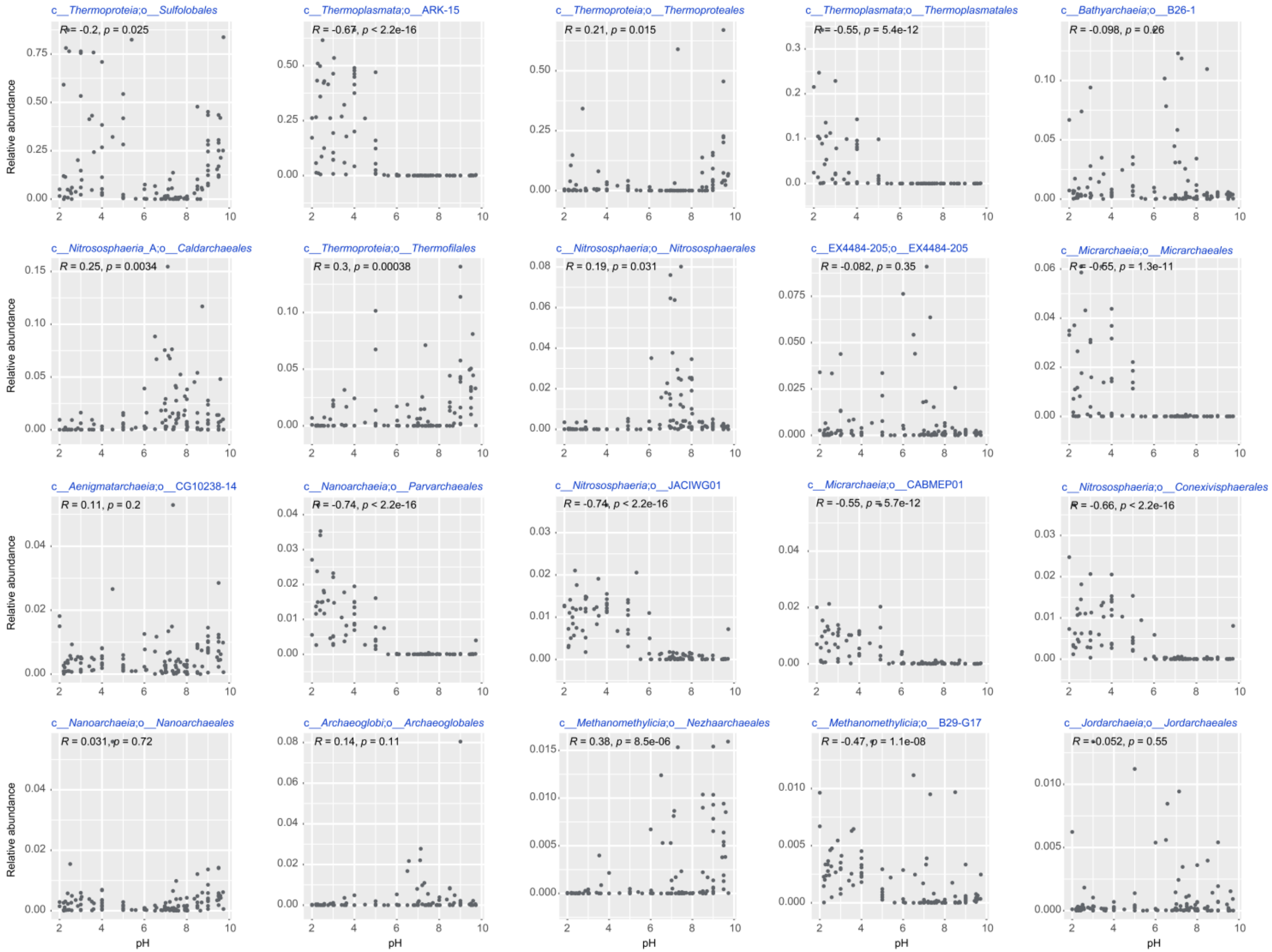

Supplementary Fig. 5 Correlation between pH and relative abundance of top 20 abundant archaeal orders in microbial community.

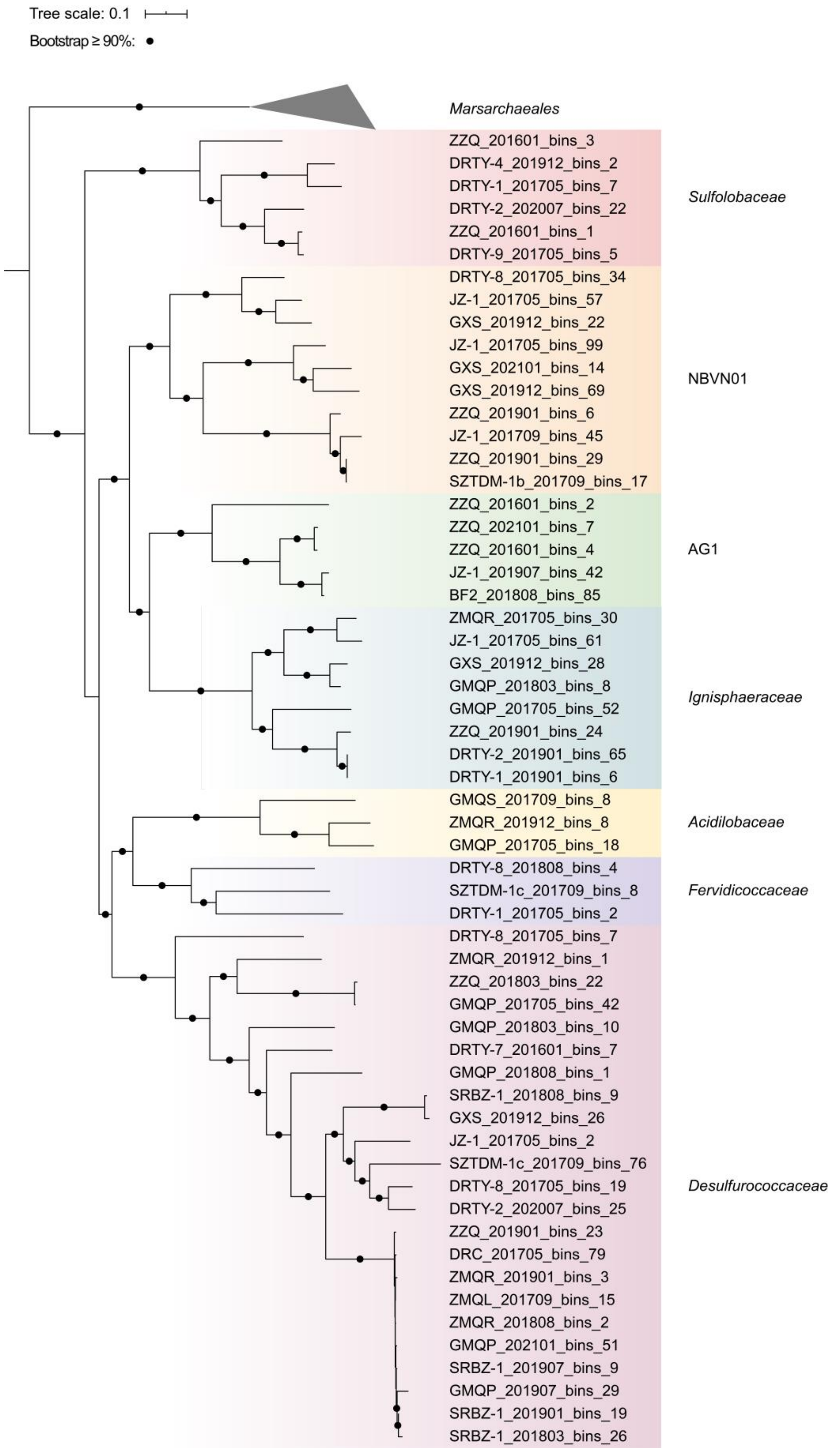

Supplementary Fig. 6 Phylogenomic tree of *Sulfolobales* with LG+F+R10 as the best model.

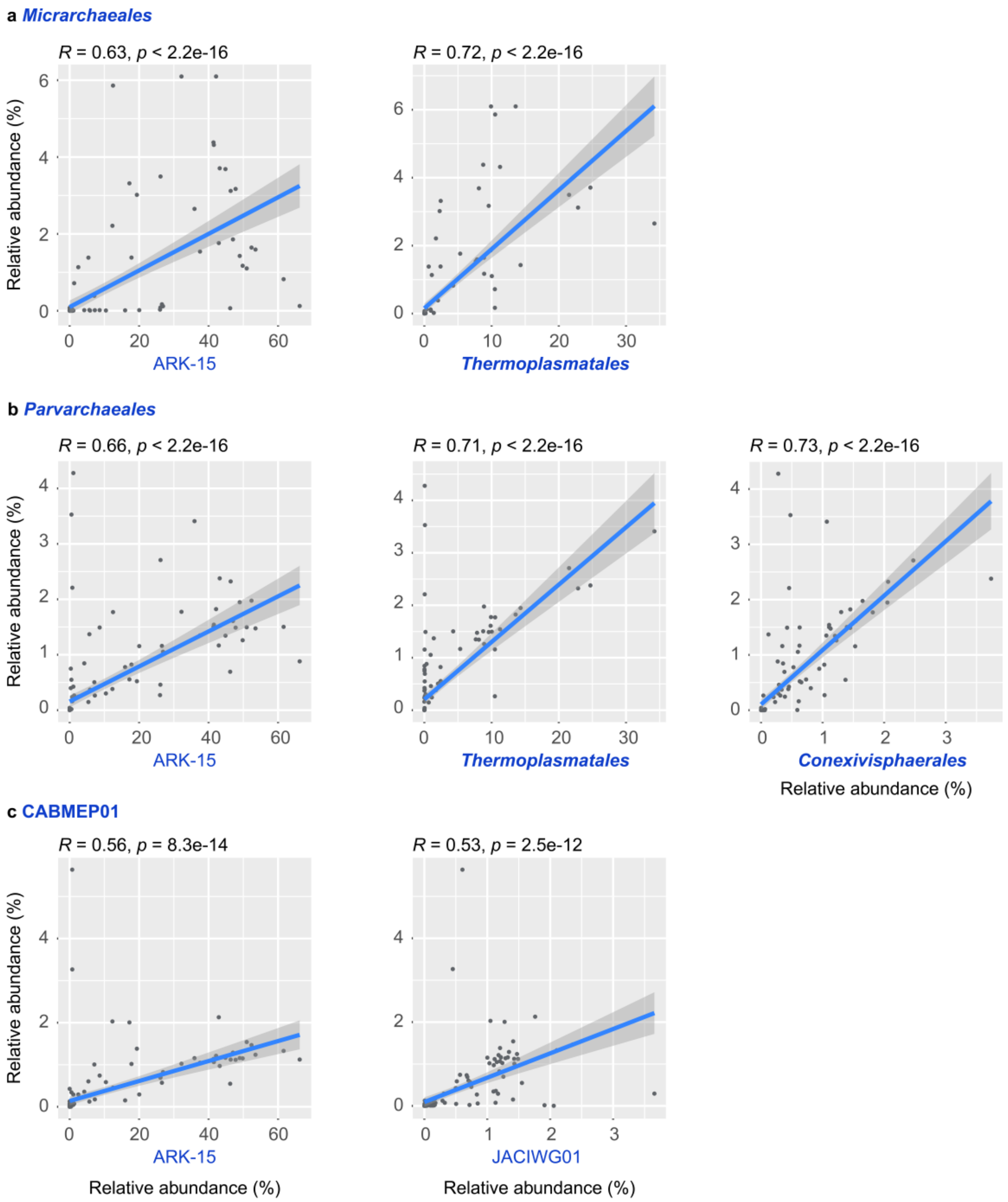

Supplementary Fig. 7. Correlation of relative abundance in microbial community between DPANN orders and other archaeal orders. (a) Correlation between the relative abundance of *Micrarchaeales* and ARK-15/*Thermoplasmatales*; (b) correlation between the relative abundance of *Parvarchaeales* and ARK-15/*Thermoplasmatales*/*Conexivisphaerales*; (c) correlation between the relative abundance of CABMEP01 and ARK-15/JACIWG01.

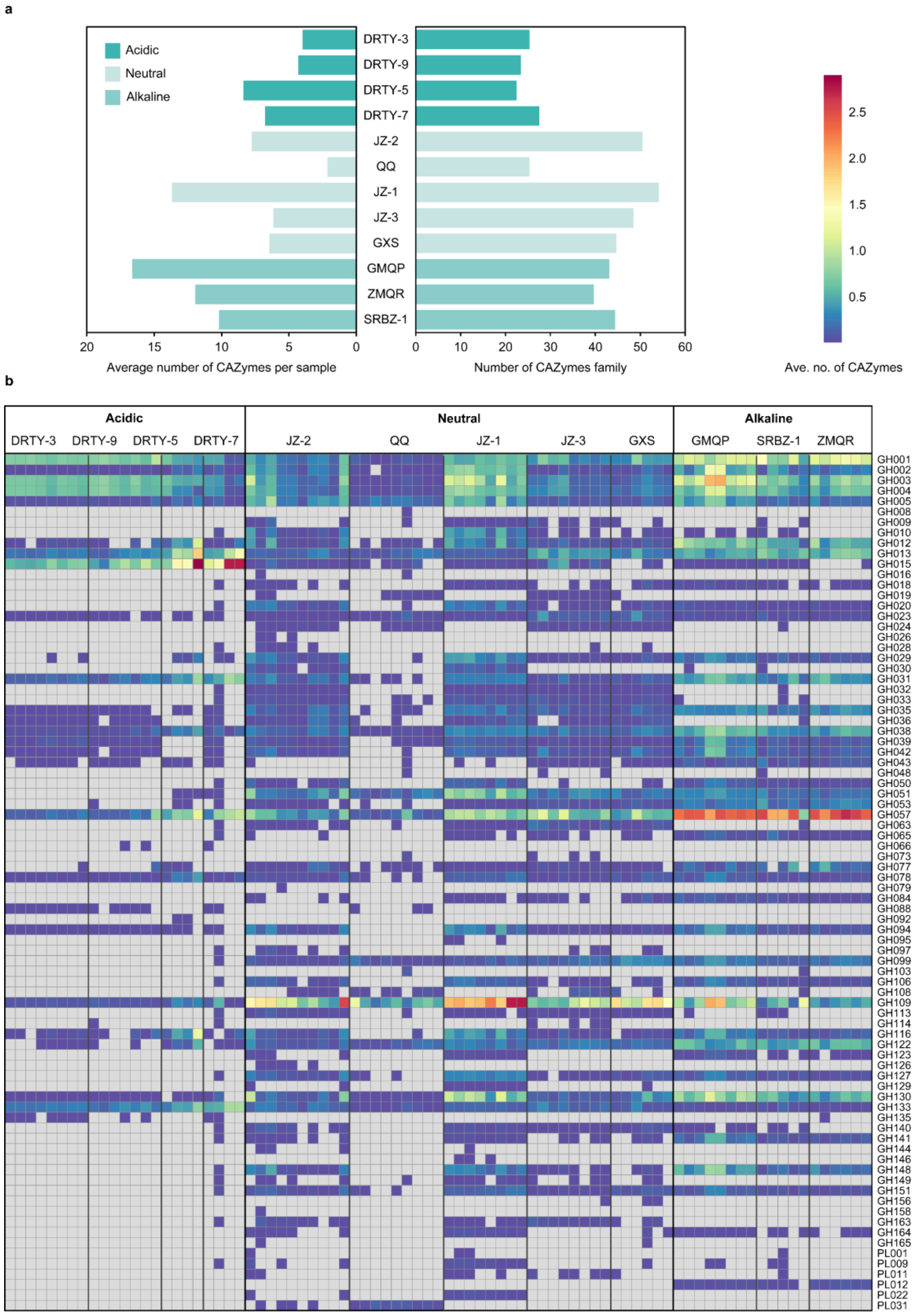

Supplementary Fig. 8 Variation of CAZymes encoded by archaeal communities in representative springs under different pH conditions. (a) Quantity of CAZymes and their respective families in acidic, neutral, and alkaline geothermal springs; (b) the average number of CAZymes in various families of glycoside hydrolase and polysaccharide lyase across geothermal springs under different pH conditions.

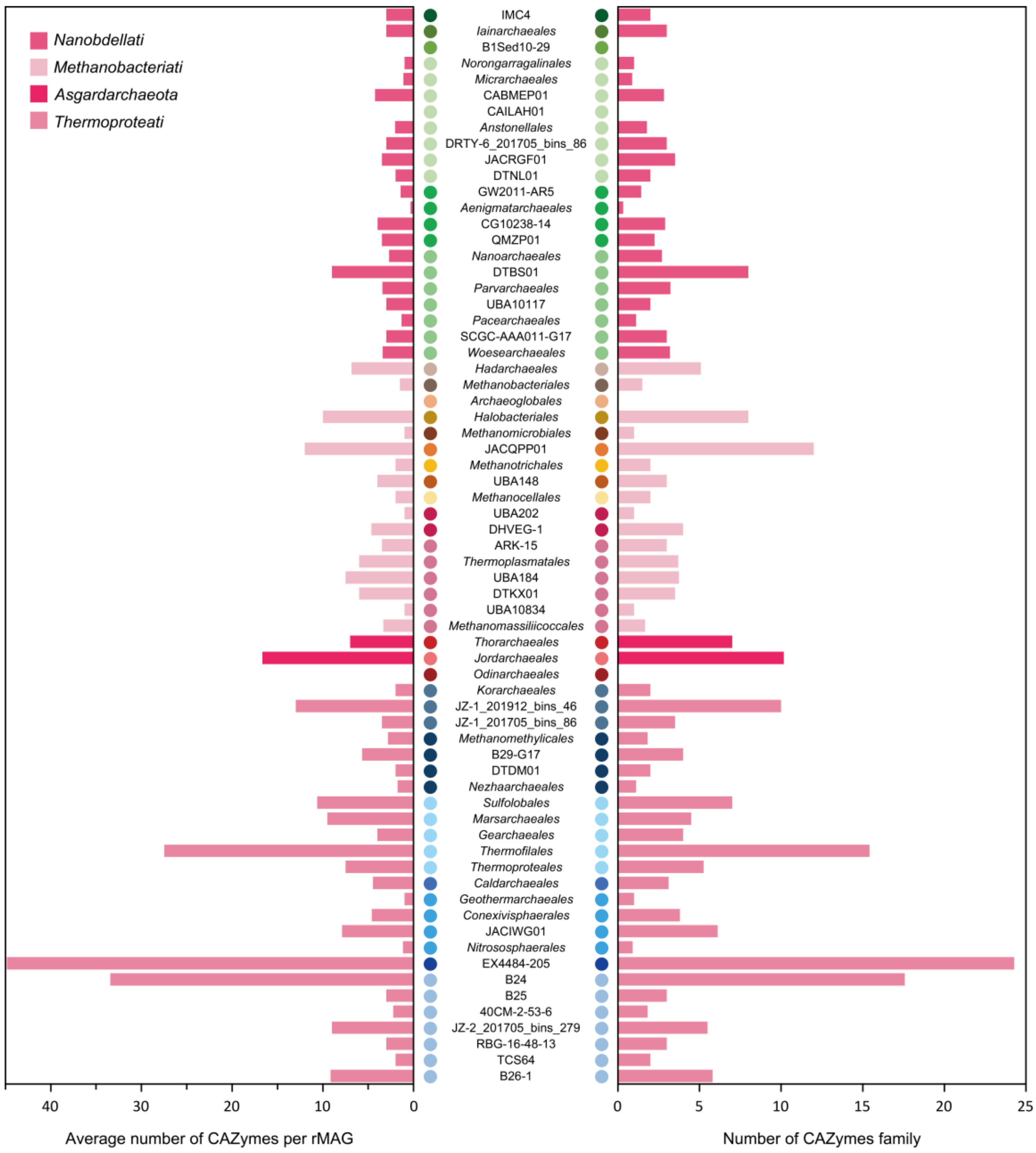

Supplementary Fig. 9 Quantity of CAZymes and their corresponding functional family in each archaeal order.

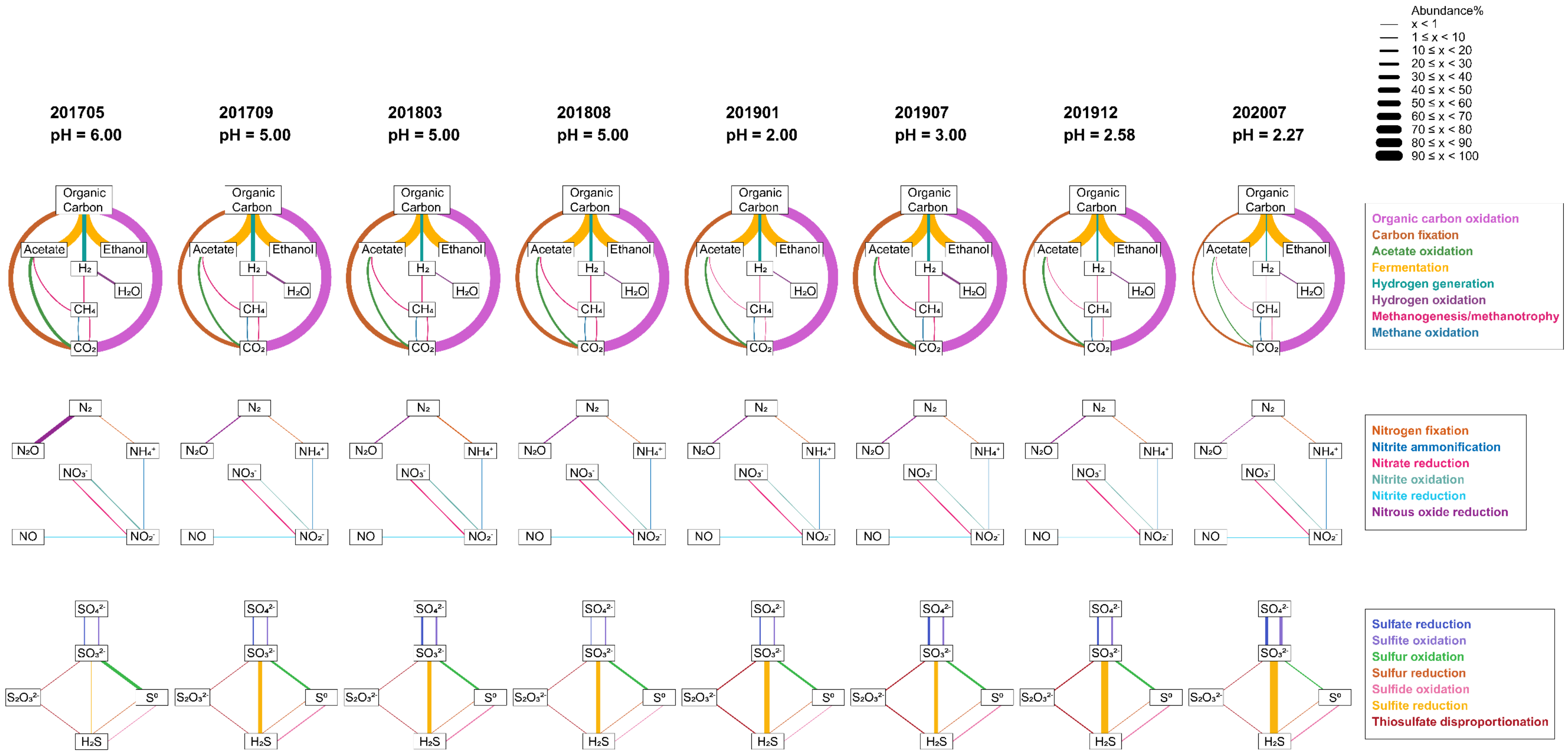

Supplementary Fig. 10 Temporal change in key biogeochemical cycles of carbon, nitrogen, sulfur and hydrogen in archaeal community from DRTY-6 spring.

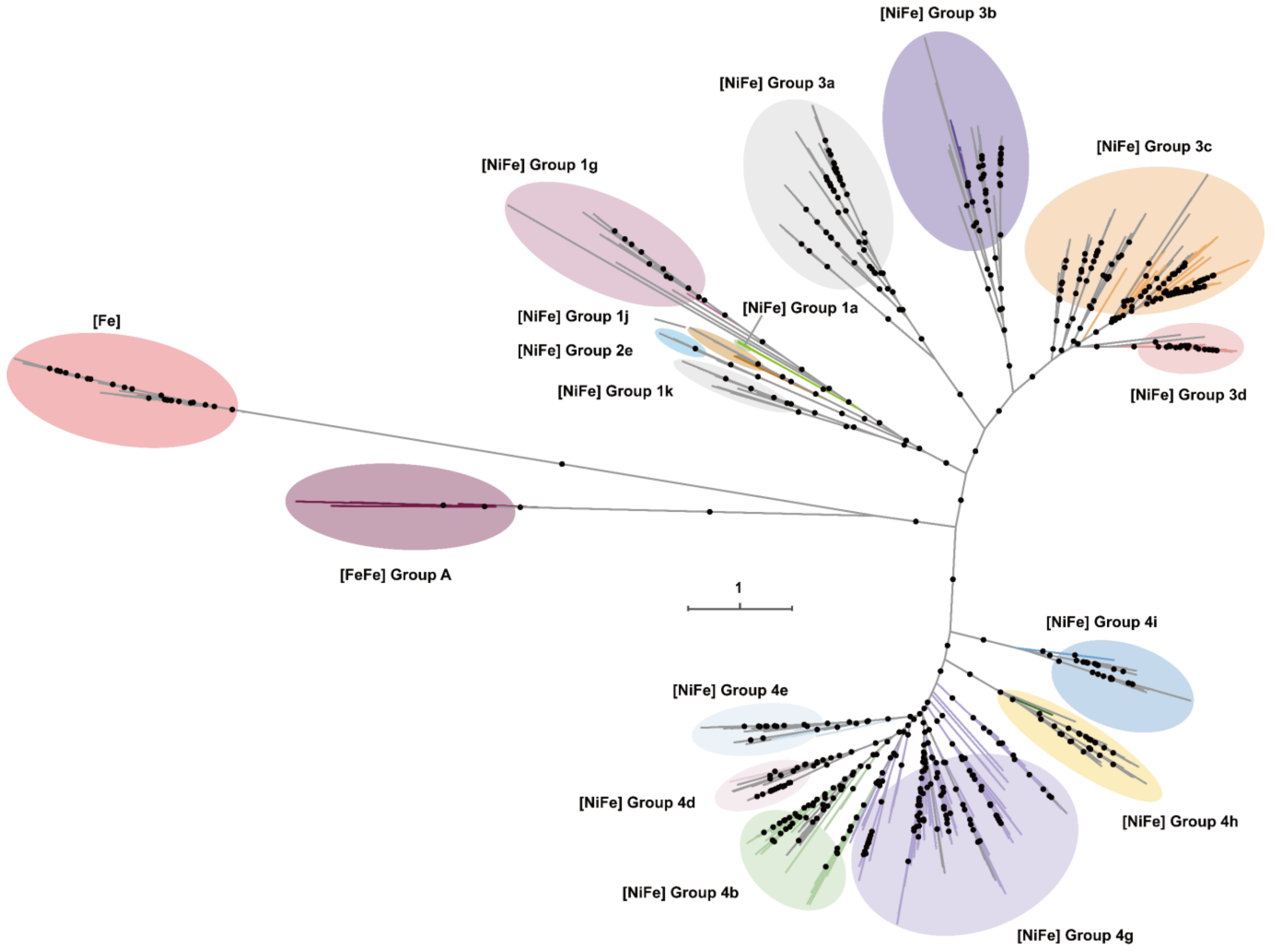

Supplementary Fig. 11 Phylogenetic tree of hydrogenase encoded by Archaea in this study and public database. The colored and gray branches represent hydrogenases derived from present study and public database, respectively.

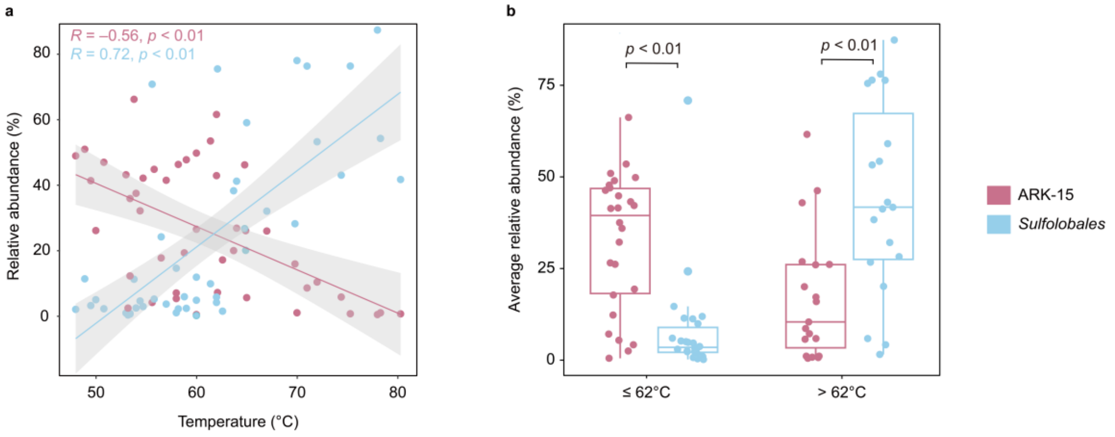

Supplementary Fig. 12 Relationship between temperature and the relative abundance of archaeal orders ARK-15 and *Sulfolobales*. (a) Correlation of relative abundance of ARK-15 and *Sulfolobales* with temperature. (b) Comparison of the relative abundance of ARK-15 and *Sulfolobales* below and above 62°C.

Supplementary Table 1. Absolute and relative abundance of Archaea determined by qPCR experiments. Samples were collected from Tengchong geothermal springs in Aug, 2023.

Sample	Microbial 16S rRNA gene (EMP-F, EMP-R)		Archaeal 16S rRNA gene (Arch519F, Arch915R)		Relative abundance of Archaea	
	Ct	Absolute copy number/ g	Ct	Absolute copy number/ g	qPCR	Metagenome
DRTY-2	23.9	3.67E+07	19.8	3.53E+07	96.4%	57.1 – 97.7% (avg. 83.1%)
DRTY-3	29.7	8.90E+05	25.3	7.00E+05	78.7%	56.4 – 87.1% (avg. 72.5%)
DRTY-9	25.5	1.31E+07	22.0	7.53E+06	57.4%	74.7 – 97.8% (avg. 86.9%)
DRTY-19	24.7	2.28E+07	21.6	1.01E+07	44.3%	34.5 – 56.9% (avg. 45.7%)
JZ-1	26.2	3.66E+08	26.2	2.85E+08	77.9%	10.1 – 58.3% (avg. 36.7%)
JZ-3	19.6	6.00E+08	18.7	7.83E+07	13.1%	0.1 – 6.9% (avg. 2.9%)
JZ-4	21.6	1.67E+08	20.6	2.05E+07	12.3%	0.2 – 7.4% (avg. 2.7%)
QQ	21.9	1.39E+08	19.7	3.67E+07	26.4%	3.1 – 20.1% (avg. 9.6%)

Supplementary Table 2. Relationship between archaeal composition and function with environmental parameters based on one-sided generalized linear model analysis with 95% confidence intervals.

rMAGs			KOs		
**Enviromental parameter**	**Chi-square (χ^2)**	***p*-value**	**Enviromental parameter**	**Chi-square (χ^2)**	***p*-value**
pH	**40.8**	**1.69E-10**	pH	**46.0**	**1.17E-11**
Temperature (°C)	**29.2**	**6.40E-08**	Temperature (°C)	**34.2**	**4.91E-09**
SO_4^{2-} (mg/kg)	**8.42**	**0.004**	SO_4^{2-} (mg/kg)	**8.18**	**0.004**
NO_2^--N (mg/kg)	**3.84**	**0.041**	NO_2^--N (mg/kg)	**4.00**	**0.045**
EC (ms/cm)	1.34	0.248	EC (ms/cm)	1.05	0.306
NO_3^--N (mg/kg)	0.82	0.364	Total organic carbon (g/kg)	0.88	0.349
Total organic carbon (g/kg)	0.35	0.552	Total N (g/kg)	0.71	0.400
Total N (g/kg)	0.34	0.561	Organic matter (g/kg)	0.58	0.447
Organic matter (g/kg)	0.33	0.563	NO_3^--N (mg/kg)	0.48	0.487
Total P (g/kg)	0.30	0.583	Total P (g/kg)	0.33	0.564
Salinity (g/kg)	0.18	0.668	Salinity (g/kg)	0.21	0.647
Cl^- (mg/kg)	0.07	0.795	NH_3-N (mg/kg)	0.08	0.777
NH_3-N (mg/kg)	0.05	0.825	Cl^- (mg/kg)	0.00	0.969
