# Supplementary material for: Analysis of nearly 3000 archaeal genomes from terrestrial geothermal springs sheds light on interconnected biogeochemical processes
Source: Nat Commun. 2024 May 14;15:4066. doi: 10.1038/s41467-024-48498-5 (PMC11094006; doi:10.1038/s41467-024-48498-5)
Supplement: Supplementary file 3 — Description of Additional Supplementary Files [file 41467_2024_48498_MOESM3_ESM.pdf]

Description of Additional Supplementary Files

Analysis of nearly 3,000 archaeal genomes from terrestrial geothermal springs sheds light on interconnected biogeochemical processes

Qi *et al.*

The PDF file includes:

Legends for Supplementary Data 1 to Data 11

File Name: Supplementary Data 1

Description: Overview of 152 samples in Tengchong geothermal springs.

File Name: Supplementary Data 2

Description: Relative abundance of 8,757 representative rpS3 proteins in microbial communities.

File Name: Supplementary Data 3

Description: Overview of 2,949 archaeal MAGs.

File Name: Supplementary Data 4

Description: Completeness estimates for 467 small Archaea MAGs based on 48 marker genes.

File Name: Supplementary Data 5

Description: Relative abundance of 603 archaeal rMAGs in microbial communities.

File Name: Supplementary Data 6

Description: Relative abundance of archaeal phyla, classes and orders in microbial communities based on rMAGs and *rpS3* genes.

File Name: Supplementary Data 7

Description: Functional annotation of 603 Archaea rMAGs ordered by key pathways and processes of interest.

File Name: Supplementary Data 8

Description: Expressed genes associated with carbon, sulfur and nitrogen metabolisms in Archaea.

File Name: Supplementary Data 9

Description: Expressed genes associated with CAZymes in Archaea.

File Name: Supplementary Data 10

Description: Classification and function for hydrogenase encoded by Archaea.

File Name: Supplementary Data 11

Description: Expressed genes associated with carbohydrate-metabolizing enzymes, peptidases and transporters in *Asgardarchaeota* and small Archaea.
