# Supplementary material for: Analysis of nearly 3000 archaeal genomes from terrestrial geothermal springs sheds light on interconnected biogeochemical processes
Source: Nat Commun. 2024 May 14;15:4066. doi: 10.1038/s41467-024-48498-5 (PMC11094006; doi:10.1038/s41467-024-48498-5)
Supplement: Supplementary file 15 — Reporting Summary [file 41467_2024_48498_MOESM15_ESM.pdf]

Corresponding author(s): heng-Shuang Hua, Brian P. Hedlund, Wen-Jun Li

Last updated by author(s): Apr 28, 2024

Reporting Summary

Nature Portfolio wishes to improve the reproducibility of the work that we publish. This form provides structure for consistency and transparency in reporting. For further information on Nature Portfolio policies, see our Editorial Policies and the Editorial Policy Checklist.

Statistics

For all statistical analyses, confirm that the following items are present in the figure legend, table legend, main text, or Methods section.

n/a	Confirmed	
☐	☒	The exact sample size (*n*) for each experimental group/condition, given as a discrete number and unit of measurement
☐	☒	A statement on whether measurements were taken from distinct samples or whether the same sample was measured repeatedly
☐	☒	The statistical test(s) used AND whether they are one- or two-sided<br>*Only common tests should be described solely by name; describe more complex techniques in the Methods section.*
☐	☒	A description of all covariates tested
☐	☒	A description of any assumptions or corrections, such as tests of normality and adjustment for multiple comparisons
☐	☒	A full description of the statistical parameters including central tendency (e.g. means) or other basic estimates (e.g. regression coefficient) AND variation (e.g. standard deviation) or associated estimates of uncertainty (e.g. confidence intervals)
☐	☒	For null hypothesis testing, the test statistic (e.g. *F*, *t*, *r*) with confidence intervals, effect sizes, degrees of freedom and *P* value noted<br>*Give P values as exact values whenever suitable.*
☐	☒	For Bayesian analysis, information on the choice of priors and Markov chain Monte Carlo settings
☐	☒	For hierarchical and complex designs, identification of the appropriate level for tests and full reporting of outcomes
☐	☒	Estimates of effect sizes (e.g. Cohen's *d*, Pearson's *r*), indicating how they were calculated

Our web collection on statistics for biologists contains articles on many of the points above.

Software and code

Policy information about availability of computer code

Data collection	No software was used for data collection.
Data analysis	Open source tools:<br>1. Metagenomic sequence assembly: SPAdes (v.3.9.0)<br>2. Sequences mapping: bbmap (v.38.92)<br>3. Genome binning: MaxBin2 (v.2.2.7), CONCOCT (v.1.1.0), MetaBAT (v.2.12.1), DASTool (v.1.1.3)<br>4. Genome bins quality evaluation: CheckM (v.1.1.3)<br>5. Genome dereplication: dRep (v.3.2.2)<br>6. Taxonomic assignment of genomes: GTDB-Tk (v.2.1.0)<br>7. Poorly alignment regions remover: TrimAl (v.1.4.rev22)<br>8. Phylogenetic tree construction: IQ-TREE (v.1.6.12)<br>9. Gene prediction: Prodigal (v.2.6.3)<br>10. Marker gene recruitment: AMPHORA (v.2.0)<br>11. Sequence dereplication: USEARCH (v.11.0.667)<br>12. Taxonomic assignment of sequence: QIIME<br>13. Functional annotation: METABOLIC (v.4.0), KofamScan (v.1.3.0)<br>14. Local alignment searching: HMMer (v.3.3.2)<br>15. Sequence alignment: MUSCLE (v.3.8.31)<br>16. Tree visualization: iTOL (v.6)

17. Quality-control of metatranscriptomic reads: Sickle (v.1.33)
18. Statistical analyses: R platform (http://cran.r-project.org)
19. Co-occurrence network: SparCC
20. Network visualization: Gephi (v.0.10.1)

For manuscripts utilizing custom algorithms or software that are central to the research but not yet described in published literature, software must be made available to editors and reviewers. We strongly encourage code deposition in a community repository (e.g. GitHub). See the Nature Portfolio guidelines for submitting code & software for further information.

Data

Policy information about availability of data

All manuscripts must include a data availability statement. This statement should provide the following information, where applicable:

- Accession codes, unique identifiers, or web links for publicly available datasets
- A description of any restrictions on data availability
- For clinical datasets or third party data, please ensure that the statement adheres to our policy

2,949 metagenome-assembled genomes of Archaea described in this study have been deposited to NCBI under the BioProject PRJNA544494: BioSample id 2,949 metagenome-assembled genomes of Archaea described in this study have been deposited to NCBI under the BioProject PRJNA544494: BioSample id SAMN18253264 to SAMN18253267, SAMN18253269, SAMN18253270, SAMN18838809, SAMN19656016 to SAMN19656018, SAMN28867992 to SAMN28867995, SAMN28867997 to SAMN28867999, SAMN31028420 to SAMN31028426, SAMN31028428 to SAMN31028439, SAMN31028763, SAMN34195732, SAMN36035244 to SAMN36035357. The accession numbers of genomes are JAVYKE000000000 to JAWCTO000000000. Supplementary data, comprising the treefile of Archaea and hydrogenase, the rpS3 protein database, and co-occurrence networks between Archaea and Bacteria, are accessible in the FigShare repository at https://doi.org/10.6084/m9.figshare.25650441. The links to the databases used in this study are listed below: CAZy database [https://www.cazy.org/]; Genome Taxonomy database [https://data.ace.uq.edu.au/public/gtdb/data/releases/release207/]; HMM database within METABOLIC program (v.4.0) [https://github.com/AnantharamanLab/METABOLIC/releases].

Research involving human participants, their data, or biological material

Policy information about studies with human participants or human data. See also policy information about sex, gender (identity/presentation), and sexual orientation and race, ethnicity and racism.

Reporting on sex and gender	N/A
Reporting on race, ethnicity, or other socially relevant groupings	N/A
Population characteristics	N/A
Recruitment	N/A
Ethics oversight	N/A

Note that full information on the approval of the study protocol must also be provided in the manuscript.

Field-specific reporting

Please select the one below that is the best fit for your research. If you are not sure, read the appropriate sections before making your selection.

☐ Life sciences ☐ Behavioural & social sciences ☒ Ecological, evolutionary & environmental sciences

For a reference copy of the document with all sections, see nature.com/documents/nr-reporting-summary-flat.pdf

Ecological, evolutionary & environmental sciences study design

All studies must disclose on these points even when the disclosure is negative.

Study description	The main point of this study is to reveal the diversity, functionality, potential roles in biogeochemical processes and geological controls on Archaea in geothermal spring ecosystems. This study involved several types of data: 1) metagenomes and metagenome-assembled genomes; 2) metatranscriptomes; 3) quantitative PCR
Research sample	Our research samples were collected from hot spring sediments in Tengchong County, Yunnan province, China. Those samples contain large quantity of archaeal cells which are the main research objects. Permits for all samples were obtained.
Sampling strategy	Hot spring sediment samples were widely collected in 50 mL sterile tubes using sterile spatulas and spoons and stored in liquid nitrogen before transporting to the lab. 152 samples from 48 different sites were identified to contain Archaea.
Data collection	Community genomic DNA was extracted from approximately 20 g of sediment material using PowerSoil DNA Isolation kit (MoBio). DNA concentrations of the extract and constructed libraries (with insert size of 350 bp) were measured with a Qubit fluorometer. Metagenomic sequence data for the two samples are generated using Illumina Hiseq 4000 instruments at Beijing Novogene

	Bioinformatics Technology Co., Ltd (Beijing, China). The amount of raw sequence data was ~30 Gbp (2×150bp) for each sample. Data was recorded by Y.L.Q..
Timing and spatial scale	Samples were collected from 2016 to 2021, once or twice a year, summer and winter. Detailed collecting date for each sample was recorded in Supplementary Data 1.
Data exclusions	No data was excluded.
Reproducibility	Locations and details for all sample collection and experiments are described in as much detail possible to promote reproducibility. No replicates were conducted since archaeal genomes are our targets. We aim to reconstruct as many archaeal genomes as possible from diverse samples rather than duplicating genomes from replicates.
Randomization	It is not relevant to our study since our study is to discover the diversity of Archaea. So only samples contain those microbes were kept.
Blinding	Mis-assembled scaffolds introduced by assemblers may result in the blinding. Also, bias might be existed during the genome binning step. To avoid this, we used several binning tools and adopted DasTool to select the best bin.

Did the study involve field work? [x] Yes [ ] No

Field work, collection and transport

Field conditions	Samples are from thermal habitats with temperature ranging from 23.0 to 100.0°C and pH ranging from 2.0 to 9.7.
Location	A total of 152 sediment samples covering 48 geothermal pools/streams located in Tengchong County (Yunnan Province, China), which located at the collision boundary between the India and Eurasia plates. Details for each location was recorded in Supplementary Data 1.
Access & import/export	The samples were collected with the permission of Yunnan Tengchong Volcano and Spa Tourist Attraction Development Corporation.
Disturbance	No disturbance.

Reporting for specific materials, systems and methods

We require information from authors about some types of materials, experimental systems and methods used in many studies. Here, indicate whether each material, system or method listed is relevant to your study. If you are not sure if a list item applies to your research, read the appropriate section before selecting a response.

Materials & experimental systems

n/a	Involved in the study
[x]	[ ] Antibodies
[x]	[ ] Eukaryotic cell lines
[x]	[ ] Palaeontology and archaeology
[x]	[ ] Animals and other organisms
[x]	[ ] Clinical data
[x]	[ ] Dual use research of concern
[x]	[ ] Plants

Methods

n/a	Involved in the study
[x]	[ ] ChIP-seq
[x]	[ ] Flow cytometry
[x]	[ ] MRI-based neuroimaging

Plants

Seed stocks	*Report on the source of all seed stocks or other plant material used. If applicable, state the seed stock centre and catalogue number. If plant specimens were collected from the field, describe the collection location, date and sampling procedures.*
Novel plant genotypes	*Describe the methods by which all novel plant genotypes were produced. This includes those generated by transgenic approaches, gene editing, chemical/radiation-based mutagenesis and hybridization. For transgenic lines, describe the transformation method, the number of independent lines analyzed and the generation upon which experiments were performed. For gene-edited lines, describe the editor used, the endogenous sequence targeted for editing, the targeting guide RNA sequence (if applicable) and how the editor was applied.*
Authentication	*Describe any authentication procedures for each seed stock used or novel genotype generated. Describe any experiments used to assess the effect of a mutation and, where applicable, how potential secondary effects (e.g. second site T-DNA insertions, mosiacism, off-target gene editing) were examined.*
